# The Transcriptome Profile of Retinal Pigment Epithelium and Müller Cell Lines Protected by Risuteganib Against Hydrogen Peroxide Stress

**DOI:** 10.1089/jop.2022.0015

**Published:** 2022-09-12

**Authors:** Zixuan Shao, Marilyn Chwa, Shari R. Atilano, John Park, Hampar Karageozian, Vicken Karageozian, M. Cristina Kenney

**Affiliations:** ^1^ Allegro Ophthalmics, LLC, San Juan Capistrano, California, USA.; ^2^ Gavin Herbert Eye Institute and University of California Irvine, Irvine, California, USA.; ^3^ Department of Pathology and Laboratory Medicine, University of California Irvine, Irvine, California, USA.

**Keywords:** retina, risuteganib, hydrogen peroxide, transcriptome, RNA-seq

## Abstract

**Purpose:**

Oxidative stress contributes to the pathogenesis of vision-impairing diseases. In the retina, retinal pigment epithelium (RPE) and Müller cells support neuronal homeostasis, but also contribute to pathological development under stressed conditions. Recent studies found that the investigational drug risuteganib (RSG) has a good safety profile, provided protection in experimental models, and improved visual acuity in patients. The present *in vitro* study evaluated the effects of RSG in RPE and Müller cell lines stressed with the oxidant hydrogen peroxide (H_2_O_2_).

**Methods:**

Human RPE (ARPE-19) and Müller (MIO-M1) cell lines were treated with various combinations of RSG and H_2_O_2_. Trypan blue assay was used to investigate the effect of compounds on cell viability. Gene expression was measured using RNA sequencing to identify regulated genes and the biological processes and pathways involved.

**Results:**

Trypan blue assay found RSG pre-treatment significantly protected against H_2_O_2_-induced cell death in ARPE-19 and MIO-M1 cells. Transcriptome analysis found H_2_O_2_ regulated genes in several disease-relevant biological processes, including cell adhesion, migration, death, and proliferation; ECM organization; angiogenesis; metabolism; and immune system processes. RSG pre-treatment modulated these gene expression profiles in the opposite direction of H_2_O_2_. Pathway analysis found genes in integrin, AP-1, and syndecan signaling pathways were regulated. Expression of selected RSG-regulated genes was validated using qRT-PCR.

**Conclusions:**

RSG protected cultured human RPE and Müller cell lines against H_2_O_2_-induced cell death and mitigated the associated transcriptome changes in biological processes and pathways relevant to the pathogenesis of retinal diseases. These results demonstrate RSG reduced oxidative stress-induced toxicity in two retinal cell lines with potential relevance to the treatment of human diseases.

## Background

Retinal diseases represent the leading causes of blindness in developed countries.^1^ Age-related macular degeneration (AMD) and diabetic retinopathy (DR) account for a majority of these cases and are characterized by a mix of pathological neovascularization, inflammation, metabolic dysregulation, and tissue degeneration.^2,3^ It has become increasingly clear that mitochondrial dysfunction and oxidative stress play key roles in the disease pathogenesis.^4–6^ Elevated reactive oxygen species (ROS) levels and evidence of oxidative damage, such as lipid peroxidation and oxidation of proteins and DNA, have been observed in AMD^7,8^ and DR^9,10^ patients. The retina is particularly vulnerable as ROS can be readily generated due to high levels of local oxygen tension, photoirradiation, metabolic activity, and reactive molecules (eg, polyunsaturated fatty acids, lipofuscin).^11,12^ When dysregulated, prolonged oxidative stress contributes to reduced cellular functionality and cell death.

The retina is an architecturally complex tissue composed of diverse cell types with distinct physiological functions. Of those, Müller glia and retinal pigment epithelial (RPE) cells maintain the neural retina homeostasis by facilitating the transport of nutrients and waste between the retina and the vascular system, recycling of light-sensing chromophores, and secretion of growth factors and antioxidants.^13–16^ Müller glia span the entire neural retina and support both neurons and cone photoreceptors, while RPE cells form a monolayer subjacent to the photoreceptors and primarily support both cone and rod photoreceptors.^13^ RPE cells also provide light absorption and filtration functionality and have high levels of antioxidants to expedite removal of resultant ROS.^15,17^ Importantly, RPE cells have enhanced capability to withstand oxidative stress and serve to protect the surrounding retinal cells from oxidative damage, functions that has been found to be diminished at old age.^18,19^

Under pathological conditions, these cells are activated to provide an initial neuroprotective role while their primary functions become dysregulated.^13,14,20,21^ Prolonged Müller and RPE dysfunction is thought to contribute to the pathological development of neovascularization, inflammation, and degeneration of the retina.

Current treatment primarily focuses on reducing neovascularization and edema with anti-VEGF drugs, but none is approved for targeting retinal degeneration. Risuteganib (RSG) is an RGD-derived (arginine–glycine–aspartate) oligopeptide first designed for binding and inhibiting integrin activity and later found to reduce integrin expression and have antioxidative and cytoprotective properties. RSG is under clinical investigation for treatment of AMD, diabetic macular edema (DME), and dry eye disease. Several clinical trials showed that RSG has a good safety profile and improved visual acuity in dry AMD and DME patients.^22,23^ In a preclinical model of human RPE cells stressed with cigarette smoke toxin, RSG treatment reduced cell death and ROS level, upregulated cytoprotective genes, and improved mitochondrial bioenergetics and metabolic activity.^24^ In RPE cells constructed to contain AMD donor mitochondria, RSG exposure reduced the expression of apoptosis and angiogenesis genes.^25^

Altogether, previous studies found that RSG can improve visual acuity, possibly through reduction of oxidative damage and reestablishment of retinal homeostasis.

Although RSG showed promising therapeutic potential, its effect on retinal cells requires further investigation. This study aimed to identify the effect of RSG and hydrogen peroxide (H_2_O_2_), a major source of ROS primarily generated during aerobic respiration,^26^ in human Müller (MIO-M1) and RPE (ARPE-19) cell lines. Cells were treated with RSG and H_2_O_2_, followed by trypan blue assay for cell viability, RNA sequencing (RNA-seq) for whole transcriptome analysis, and quantitative real-time PCR (qRT-PCR) for validation of gene expression. The combined exposure to both RSG and H_2_O_2_ reveals the drug's therapeutic effects in the context of H_2_O_2_-induced oxidative stress. A better understanding of the drug mechanism could lead to the development of novel therapies for these debilitating diseases.

## Methods

### Cell culture reagents and conditions

The human Müller cell line MIO-M1^27^ was kindly provided by Professor Astrid Limb (University College London). Cells were cultured in Dulbecco's modified Eagle's medium (DMEM) with 4.5 g/L glucose, glutaGRO (Corning Cellgro, Manassas, VA), 10% fetal bovine serum (FBS; Sigma, St. Louis, MO), penicillin 100 U/mL, and streptomycin sulfate 0.1% mg/mL (Omega Scientific, Inc., Tarzana, CA). ARPE-19 cells^28^ were obtained from ATCC (CRL-2302; Manassas, VA) and cultured in DMEM mixture 1:1 Ham's F-12 medium (Corning, Manassas, VA), with 10% FBS, penicillin 100 U/mL, streptomycin sulfate 0.1% mg/mL, gentamicin 10 mg/mL, and amphotericin B 2.5 mg/mL (Omega Scientific, Inc.). Allegro Ophthalmics (San Juan Capistrano, CA) provided RSG; H_2_O_2_ was purchased from Sigma. Upon receipt, the cells were split 5 times to generate frozen cell stocks.

All experiments used cells at passage 5; cells were cultured for 48 h, then counted and plated at a density of 0.5 × 10^6^ cells per well, following by another 24-h culture before treatment. Cells were at subconfluent density at the onset of treatment as confluent cells have been found to be significantly more resistant to oxidative stress.^29^

### Trypan blue assay

ARPE-19 (*n* = 3) and MIO-M1 (*n* = 8–9) cells in 6-well plates were treated according to the following 5 regimens: (1) untreated control, (2) RSG for 36 h, (3) untreated for 24 h, then H_2_O_2_ for 12 h, (4) RSG pretreatment for 24 h, then H_2_O_2_ for 12 h, and (5) RSG for 24 h, then RSG and H_2_O_2_ cotreatment for 12 h; conditions labeled as control, RSG, H_2_O_2_, H_2_O_2_ + RSG pretreat, and H_2_O_2_ + RSG cotreat, respectively.

Four hundred micromolar of RSG was used because it is the cell culture equivalence of the clinical dose (1.0 mg injection into 4 mL of vitreous volume).^22^ One hundred micromolar of H_2_O_2_ was used as this concentration was found to induce moderate cytotoxicity in ARPE-19^30^ and MIO-M1^31^ cells, which simulates the elevated oxidative stress that leads to progressive cell death in the relevant retinal diseases where RSG is currently being investigated. After exposure, cells were incubated for 48 h in fresh media before cell harvest, and cell viability measurement by trypan blue assay with an automated ViCell analyzer (Beckman Coulter, Inc., Fullerton, CA). The density of viable cells was measured and normalized to mean of control as 100%.

### RNA-seq sample preparation and analysis

ARPE-19 (*n* = 6) and MIO-M1 (*n* = 6) cells in 6-well plates were treated according to the following 3 regimens: (1) untreated control, (2) untreated for 24 h, then H_2_O_2_ for 12 h, and (3) RSG pretreatment for 24 h, then H_2_O_2_ for 12 h; conditions labeled as control, H_2_O_2_, and H_2_O_2_ + RSG, respectively. Four hundred micromolar of RSG and 100 μM of H_2_O_2_ were used. After exposure, cells were incubated for 48 h in fresh media before cell collection. Total RNA was extracted using the RNeasy Mini Kit (Qiagen, Hilden, Germany), and DNA was removed with TURBO DNA-free (Thermo Fisher Scientific, Waltham, MA). RNA quality was measured with Bioanalyzer (Agilent Genomics, Santa Clara, CA).

RNA-seq libraries were prepared using the NEBNext Ultra RNA Library Prep Kit (New England Biolabs, Ipswich, MA) and sequenced on HiSeq 2500 (Illumina, San Diego, CA) to generate 10–15 million single-end, 100 base-pair reads per sample. RNA-seq reads were quality tested by FASTQC,^32^ and then aligned by STAR^33^ to human genome (GRCh38.p12) and transcriptome (GENECODE v30) references, followed by read quantification using featureCounts.^34^

Principal component analysis (PCA) was used to visualize the RNA-seq data set along the top 3 dimensions that captured the most variance, using the top 15,000 expressed genes calculated by the DESeq2's VST method.^35^ Differential expression analysis was performed using edgeR^36^ with analysis limited to genes with count per million of 4 or greater in at least 6 samples (referred to as expressed genes). Differentially expressed (DE) genes have a false discovery rate (FDR) <0.05 and are either upregulated or downregulated based on signs of log2-fold change (log2FC).

DE genes were submitted to goseq^37^ for enrichment of gene ontology biological processes^38,39^ and NCI Nature biological pathways^40^ that are overrepresented in the gene list. Biological processes or pathways with FDR <0.05 were considered to be enriched, that is, significantly overrepresented in the list of DE genes. Enriched biological processes were condensed and visualized with REVIGO^41^ with similarity metric set to small; selected representative processes were labeled. Biological processes were grouped into categories by first summarizing those with an FDR <0.001 using REVIGO, and then a manual review of the results to form categories. Heatmaps were used to visualize gene expression levels, which were averaged by condition and then normalized to mean expression across all samples; genes were clustered by Euclidean distance.^42^ ERSSA evaluates whether a sufficient sample size was used; analysis was performed with filter cutoff = 4, log2FC cutoff = 0.25, and 50 subsamples per replicate level.^43^

### Quantitative real-time PCR

ARPE-19 (*n* = 6) and MIO-M1 (*n* = 6) cells in 6-well plates were used for qRT-PCR. Three hundred nanograms of RNA was reverse transcribed into cDNA with SuperScript IV VILO Master Mix (Thermo Fisher, Waltham, MA) on a ProFlex PCR system (Thermo Fisher). qRT-PCR was performed with PowerUp SYBR Green Master Mix on a QuantStudio 5 Real-Time PCR System. Predesigned SYBR Green primers were used (KiCqStart SYBR Green Primers [Sigma, Burlington, MA] and QuantiTect Primer Assays [Qiagen], Table 1). *HPRT1* was the reference gene used.

**Table 1.** Description of Genes Analyzed by Quantitative Real-Time Polymerase Chain Reaction

Cell	Gene symbol	Gene name	GenBank accession number	Function	Company	Catalog number
ARPE-19	*FOS*	Fos proto-oncogene, AP-1 transcription factor subunit	NM_005252	Cell proliferation & death	Sigma	H_FOS_1
ARPE-19	*EGR1*	Early growth response 1	NM_001964	Cell proliferation & death	Sigma	H_EGR1_1
ARPE-19	*SGK1*	Serum/glucocorticoid-regulated kinase 1	NM_001143676	Cell proliferation & death	Sigma	H_SGK1_1
ARPE-19	*FAIM*	Fas apoptotic inhibitory molecule	NM_001033030	Cell proliferation & death	Sigma	H_FAIM_1
ARPE-19	*HBEGF*	Heparin binding EGF-like growth factor	NM_001945	Cell proliferation & death	Sigma	H_HBEGF_1
ARPE-19	*PDGFA*	Platelet-derived growth factor subunit A	NM_002607	Cell proliferation & death	Sigma	H_PDGFA_1
ARPE-19	*POSTN*	Periostin	NM_001135934	ECM, cell adhesion, & migration	Sigma	H_POSTN_1
ARPE-19	*CCN2/CTGF*	Cellular communication network factor 2	NM_001901	ECM, cell adhesion, & migration	Sigma	H_CTGF_1
ARPE-19	*NEDD9*	Neural precursor cell expressed, developmentally downregulated 9	NM_001142393	ECM, cell adhesion, & migration	Sigma	H_NEDD9_1
ARPE-19	*CCN1/CYR61*	Cellular communication network factor 1	NM_001554	ECM, cell adhesion, & migration	Qiagen	QT00003451
ARPE-19	*THBS1*	Thrombospondin 1	NM_003246	ECM, cell adhesion, & migration	Qiagen	QT00028497
ARPE-19	*ADAMTS9*	ADAM metallopeptidase with thrombospondin-type 1 motif 9	NM_182920	ECM, cell adhesion, & migration	Sigma	H_ADAMTS9_1
MIO-M1	*BACH2*	BTB domain and CNC homolog 2	NM_001170794	Cell proliferation & death	Sigma	H_BACH2_1
MIO-M1	*MEGF10*	Multiple EGF-like domains 10	NM_001256545	Cell proliferation & death	Sigma	H_MEGF10_1
MIO-M1	*BMF*	BCL2 modifying factor	NM_001003940	Cell proliferation & death	Sigma	H_BMF_1
MIO-M1	*EGR1*	Early growth response 1	NM_001964	Cell proliferation & death	Sigma	H_EGR1_1
MIO-M1	*SGK1*	Serum/glucocorticoid regulated kinase 1	NM_001143676	Cell proliferation & death	Sigma	H_SGK1_1
MIO-M1	*PGF*	Placental growth factor	NM_001207012	Cell proliferation & death	Sigma	H_PGF_1
MIO-M1	*COL1A1*	Collagen type I alpha 1 chain	NM_000088	ECM, cell adhesion, & migration	Qiagen	QT00037793
MIO-M1	*THSD7A*	Thrombospondin type 1 domain containing 7A	NM_015204	ECM, cell adhesion, & migration	Sigma	H_THSD7A_1
MIO-M1	*COL6A3*	Collagen type VI alpha 3 chain	NM_004369	ECM, cell adhesion, & migration	Sigma	H_COL6A3_1
MIO-M1	*FMN1*	Formin 1	NM_001103184	ECM, cell adhesion, & migration	Sigma	H_FMN1_1
MIO-M1	*COL13A1*	Collagen type XIII alpha 1 chain	NM_001130103	ECM, cell adhesion, & migration	Sigma	H_COL13A1_1
MIO-M1	*MMP3*	Matrix metallopeptidase 3	NM_002422	ECM, cell adhesion, & migration	Sigma	H_MMP3_1
ARPE-19 & MIO-M1	*HPRT1*	Hypoxanthine-guanine phosphoribosyltransferase	NM_000194	Reference gene	Sigma	H_HPRT1_1
ARPE-19 & MIO-M1	*RLBP1/CRALBP*	Retinaldehyde-binding protein 1	NM_000326	RPE cell marker	Sigma	H_RLBP1_1
ARPE-19 & MIO-M1	*OTX2*	Orthodenticle homeobox 2	NM_021728	RPE cell marker	Sigma	H_OTX2_1
ARPE-19 & MIO-M1	*PMEL/PMEL17*	Premelanosome protein	NM_006928, NM_001200054	RPE cell marker	Qiagen	Hs_PMEL_1_SG
ARPE-19 & MIO-M1	*GFAP*	Glial fibrillary acidic protein	NM_002055	Müller cell marker	Sigma	H_GFAP_1
ARPE-19 & MIO-M1	*GLUL*	Glutamate-ammonia ligase	NM_001033044	Müller cell marker	Sigma	H_GLUL_1
ARPE-19 & MIO-M1	*SLC1A3*	Solute carrier family 1 member 3	NM_001166696	Müller cell marker	Sigma	H_SLC1A3_1

### Data availability

The RNA-seq raw data are available at NCBI SRA database (accession PRJNA723610). RNA-seq analysis scripts are available at https://github.com/zshao1/RSG_H2O2. All other relevant data are within the article and its supplementary figures and tables.

### Statistical analysis

Trypan blue data are subjected to 1-way ANOVA test with multiple comparison correction by the Benjamini, Krieger, and Yekutieli method using GraphPad Prism (Version 9.0, San Diego, CA). The *q* value is defined to be the adjusted *P* value from the ANOVA test (ie, *P* value after multiple comparison correction). A *q* value ≤0.05 is considered statistically significant. qRT-PCR fold values were calculated using the 2^−ΔΔCt^ formula.^44^

## Results

### RSG pretreatment reduced cell viability loss

**FIG. 1.**
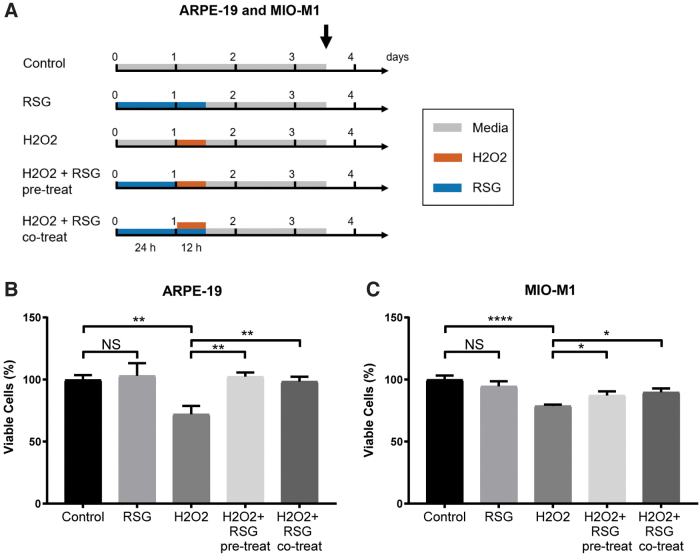
RSG protected against H_2_O_2_-induced cell viability reduction. **(A)** ARPE-19 (*n* = 3) and MIO-M1 (*n* = 8–9) cells in 6-well plates were treated according to the 5 treatment regimens for 3.5 days before cell collection and trypan *blue* assay for cell viability. [H_2_O_2_ + RSG cotreat] group received 24-h RSG pretreatment and then RSG and H_2_O_2_ cotreatment for 12 h. *Black arrow* indicates cell collection time. In ARPE-19 **(B)** and MIO-M1 **(C)** cells, RSG alone did not alter cell viability, while H_2_O_2_ significantly reduced cell viability. RSG pretreatment with and without cotreatment both significantly protected against H_2_O_2_-induced cell viability loss. Mean ± standard error of mean was plotted; NS = not significant, *q ≤ 0.05, **q ≤ 0.01, ****q ≤ 0.0001. RSG, risuteganib.

ARPE-19 and MIO-M1 cells used in this study were confirmed to express RPE cell markers (*RLBP1/CRALBP*, *OTX2*, and *PMEL/PMEL17*)^45^ and Müller cell markers (*GFAP*, *GLUL*, and *SLC1A3*),^46^ respectively (Supplementary Fig. S8 and Supplementary Table S1). Trypan blue assay was used to investigate the effect of H_2_O_2_ and RSG on cell viability (Fig. 1A).

In both cell lines, RSG treatment had no effect, while H_2_O_2_ significantly reduced cell viability to 0.72 × (*q* = 0.004) and 0.79 × of control (*q* < 0.0001) in ARPE-19 and MIO-M1 cells, respectively (Fig. 1B, C). In cells pretreated with RSG for 24 h before H_2_O_2_ exposure, cell viability was significantly improved to 1.03 × (*q* = 0.0044) and 0.87 × of control (*q* = 0.043) in ARPE-19 and MIO-M1 cells, respectively. Similarly, in cells pretreated with RSG followed by cotreatment with both RSG and H_2_O_2_, cell viability was significantly improved to 0.99 × (*q* = 0.004) and 0.90 × of control (*q* = 0.020) in ARPE-19 and MIO-M1 cells, respectively.

### RNA-seq revealed transcriptome changes by H_2_O_2_ and RSG

**FIG. 2.**
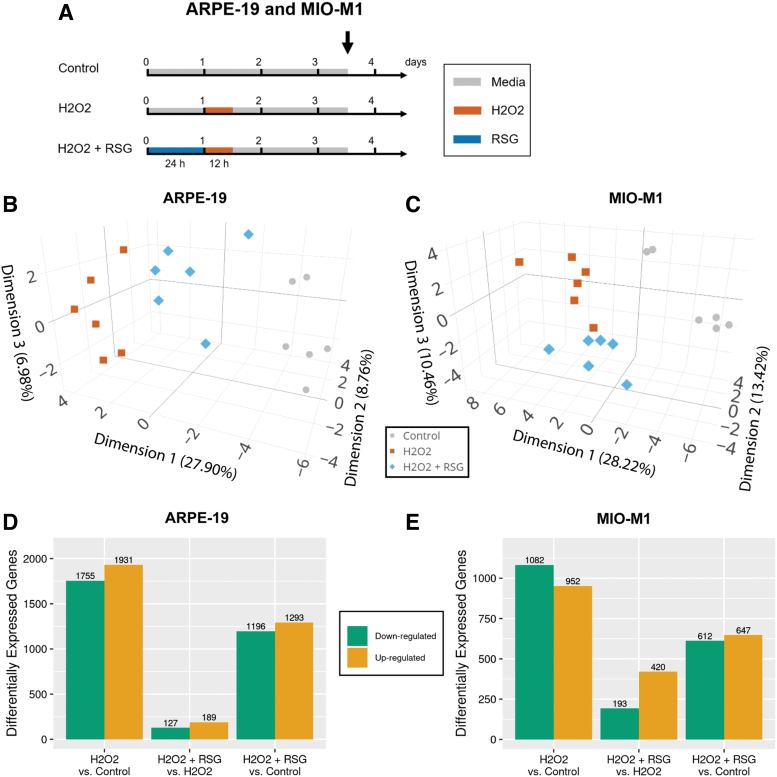
Transcriptome analysis of cells after H_2_O_2_ exposure and RSG pretreatment. **(A)** ARPE-19 (*n* = 6) and MIO-M1 (*n* = 6) cells in 6-well plates were treated according to the 3 treatment regimens for 3.5 days before cell collection and RNA-seq. *Black arrow* indicates cell collection time. **(B, C)** Principal component analysis was used to visualize the samples on the first 3 dimensions that captured the largest amount of variance (shown in axis label) in the transcriptome data set. In both ARPE-19 **(B)** and MIO-M1 **(C)** cells, samples clustered by treatment condition. **(D, E)** edgeR software was used to identify genes that are DE between the conditions. Number of DE genes are shown, separated by upregulated (*orange*) or downregulated (*green*) genes. In both ARPE-19 **(D)** and MIO-M1 **(E)** cells, H_2_O_2_ regulated substantially more genes than RSG pretreatment. RSG pretreatment reduced the effect of H_2_O_2_ on the transcriptome.

RNA-seq was used to probe the associated global transcriptome profile in control, H_2_O_2_, and RSG pretreatment + H_2_O_2_ conditions (Fig. 2A). Treatment with RSG alone was not evaluated because of the following: (1) it had a nonsignificant effect on cell viability and (2) previous study found it had a minimal effect on the transcriptome of human RPE cells.^24^ RSG pretreatment followed by RSG and H_2_O_2_ cotreatment was also not evaluated since both RSG pretreatment regimens showed comparable protection. All samples had excellent RNA quality and sequencing quality (Supplementary Table S1). PCA-based visualization of the RNA-seq data set showed distinct separation by condition in both ARPE-19 (Fig. 2B) and MIO-M1 (Fig. 2C) cells, indicative of changes in the transcriptome after H_2_O_2_ exposure and RSG pretreatment.

Differential expression analysis was performed to identify the DE genes that can be contributed to H_2_O_2_ exposure and RSG pretreatment. In both cell models, H_2_O_2_ exposure is associated with more DE genes (H_2_O_2_ vs. control; 3,686 in APRE-19 and 2,034 in MIO-M1) than RSG pretreatment (H_2_O_2_ + RSG vs. H_2_O_2_; 316 in ARPE-19 and 613 in MIO-M1), Fig. 2D, E, and Supplementary Fig. S1. In addition, cells pretreated with RSG had fewer DE genes (H_2_O_2_ + RSG vs. control; 2,489 in ARPE-19 and 1,259 in MIO-M1) compared with those with no treatment before H_2_O_2_ exposure (H_2_O_2_ vs. control).

Next, ERSSA confirmed that a sufficient sample size was used in all comparisons to produce a meaningful number of DE genes. As sample size approached *n* = 6, the number of biological replicates in this study, the average discovery trend plateaued in all comparisons (Supplementary Fig. S2). Additional samples are unlikely to significantly improve the current differential expression discovery.

### RSG suppressed transcriptome changes by H_2_O_2_

**FIG. 3.**
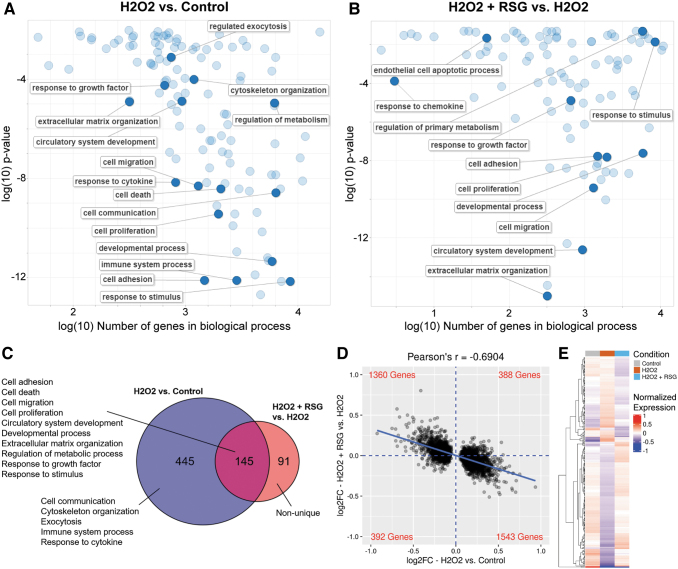
RSG pretreatment suppressed H_2_O_2_-induced expression changes in ARPE-19 cells. **(A, B)** Biological processes enriched with DE genes regulated either by H_2_O_2_ **(A)** or by RSG pretreatment **(B)** in ARPE-19 cells. Enriched processes are summarized and displayed; selected representative processes are labeled. H_2_O_2_ exposure and RSG pretreatment regulated many of the same processes. **(C)** Venn diagram represents the overlap in the biological processes enriched with DE genes. Sixty-one percent (145/236) of processes enriched with RSG pretreatment-regulated genes were also found with H_2_O_2_-regulated genes. Enriched processes can be generally grouped into several major categories as listed. **(D)** Visualization of fold change (log2FC) of DE genes regulated by H_2_O_2_. *x*-axis represents the gene's log2FC as regulated by H_2_O_2_, and *y*-axis represents the corresponding log2FC as regulated by RSG pretreatment. Display is limited to (−1.0, 1.0) range on both axes. Linear regression of data is shown as a *solid blue line*. *Blue dashed lines* separate the data into 4 quadrants with the number of genes in each quadrant labeled at the corners in *red*. A negative Pearson's correlation coefficient was observed between expression changes associated with H_2_O_2_ exposure and RSG pretreatment. **(E)** Heatmap displays the expression profile of RSG pretreatment-regulated genes across the conditions. Expression is normalized to mean expression level across all samples. Genes are ordered based on clustering analysis as shown by dendrogram on the *left*. RSG-pretreated condition appeared to resemble the control condition more than the H_2_O_2_-treated condition.

Biological processes that are overrepresented (ie, statistically enriched) in the DE genes were identified. In ARPE-19 cells, 590 and 236 biological processes were enriched with H_2_O_2_- and RSG pretreatment-regulated genes, respectively (condensed and visualized in Fig. 3A, B). Of the 236 processes enriched with RSG pretreatment-regulated genes, a majority (61%, 145/236) were also enriched with H_2_O_2_-regulated genes (Fig. 3C). The enriched processes were further grouped into major categories, which found that H_2_O_2_ uniquely modulated processes related to cell communication, cytoskeleton organization, exocytosis, the immune system, and response to cytokines. Significantly, both H_2_O_2_ and RSG pretreatment regulated genes involved in cell adhesion, migration, death, and proliferation; angiogenesis; development; extracellular matrix (ECM) organization; metabolism; and response to growth factor (GF) and stimulus.

The strong overlap prompted a closer evaluation of the gene expression changes. When all 3,686 genes regulated by H_2_O_2_ were evaluated, there was a notable inverse relationship (*r* = −0.6904) between the expression changes associated with H_2_O_2_ exposure and RSG pretreatment (Fig. 3D). The same inverse expression profile was observed in each of the biological process categories regulated by both the H_2_O_2_ and RSG pretreatments (Supplementary Fig. S3). In addition, the expression level of RSG pretreatment-regulated genes matched more closely with the control condition than the H_2_O_2_ condition (Fig. 3E).

Lastly, an analysis of the overlap in the DE genes showed that almost all (95%, 208/219) of the genes regulated by both the H_2_O_2_ and RSG pretreatments had expression changes in opposite directions, that is, genes upregulated by H_2_O_2_ exposure were downregulated by RSG pretreatment and vice versa (Table 2). Among them, genes with the largest expression changes are involved in cell proliferation and death (*FOS*, *FOSB*, *EGR1*, *CTGF*, *PDGFA*, and *FAIM*), stress response (*SGK1*), and cell adhesion and migration (*CYR61*, *THBS1*, *COL4A1*, *COL5A1*, *POSTN*, and *ADAMTS9*). RSG pretreatment also upregulated 24/27 (88.9%) of “wound healing” genes, which are involved in restoring integrity to a damaged tissue following an injury (Supplementary Fig. S7A).

**Table 2.** Differentially Expressed Genes in ARPE-19 Cells

Number of DE genes^a^		H_2_O_2_ vs. control			
		Up	Down	No change	Total
H_2_O_2_ + RSG vs. H_2_O_2_	Up	7	119	63	189
	Down	89	4	34	127
	No change	1835	1632		
	Total	1931	1755		

^a^ Genes upregulated, downregulated, or not DE are labeled as *up*, *down*, or *no change*, respectively.

DE, differentially expressed; RSG, risuteganib.

**FIG. 4.**
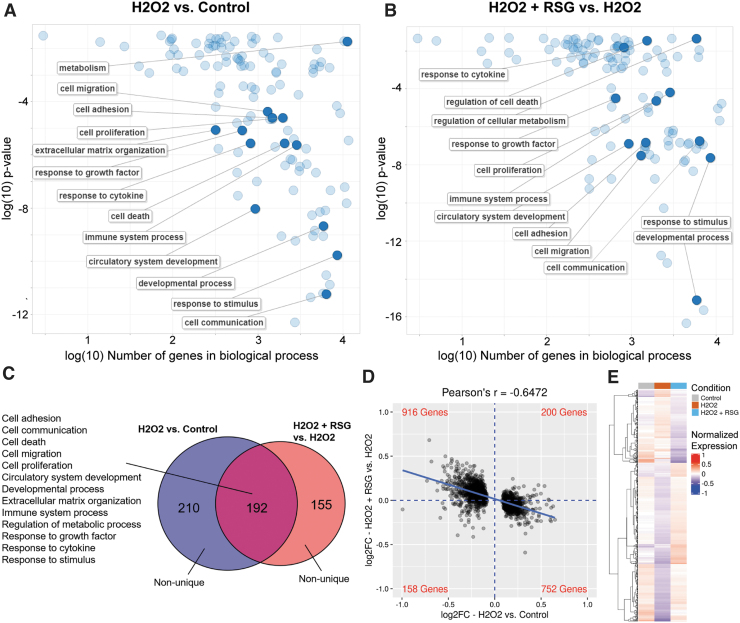
RSG pretreatment suppressed H_2_O_2_-induced expression changes in MIO-M1 cells. **(A, B)** Biological processes enriched with DE genes regulated either by H_2_O_2_ **(A)** or by RSG pretreatment **(B)** in MIO-M1 cells. Enriched processes are summarized and displayed; selected representative processes are labeled. H_2_O_2_ exposure and RSG pretreatment regulated many of the same processes. **(C)** Venn diagram represents the overlap in the biological processes enriched with DE genes. Fifty-five percent (192/347) of processes enriched with RSG pretreatment-regulated genes were also found with H_2_O_2_-regulated genes. Enriched processes can be generally grouped into several major categories as listed. **(D)** Visualization of fold change (log2FC) of DE genes regulated by H_2_O_2_. *x*-axis represents the gene's log2FC as regulated by H_2_O_2_, and *y*-axis represents the corresponding log2FC as regulated by RSG pretreatment. Display is limited to (−1.0, 1.0) range on both axes. Linear regression of data is shown as a *solid blue line*. *Blue dashed lines* separate the data into 4 quadrants, with the number of genes in each quadrant labeled at the corners in *red*. A negative Pearson's correlation coefficient was observed between expression changes associated with H_2_O_2_ exposure and RSG pretreatment. **(E)** Heatmap displays the expression profile of RSG pretreatment-regulated genes across the conditions. Expression is normalized to mean expression level across all samples. Genes are ordered based on clustering analysis as shown by dendrogram on the *left*. RSG-pretreated condition appeared to resemble control condition more than H_2_O_2_-treated condition.

A comparable expression profile was observed in MIO-M1 cells. In this study, 402 biological processes were enriched with H_2_O_2_-regulated genes and 347 with RSG pretreatment-regulated genes, of which 55% (192/347) were enriched by both (Fig. 4A–C). When biological processes were grouped into categories, 13 major categories were found to be regulated by both treatments, including cell adhesion, migration, communication, death, and proliferation; angiogenesis; development; ECM organization; immune system process; metabolism; and response to GF, cytokine, and stimulus. Evaluation of the 2,034 genes regulated by H_2_O_2_ showed an inverse relationship (*r* = −0.6472) between the expression changes associated with the 2 treatments (Fig. 4D).

Analysis of each of the 13 biological process categories showed the same inverse correlation in expression changes (Supplementary Fig. S4). Similarly, the expression level of RSG pretreatment-regulated genes matched more closely with the untreated control than the H_2_O_2_ condition (Fig. 4E). Lastly, an overlap in DE genes showed that a majority (93%, 264/284) of them had expression changes in opposite directions (Table 3). Among them, genes with largest expression changes are involved in cell proliferation and death (*BMF*, *MEGF10*, *BACH2*, and *TEAD4*), angiogenesis (*THSD7A*), and cell adhesion and migration (*FMN1*, *MYO1*, *SYNE1*, *SYNE2*, *HMCN*, *MACF1*, *COL12A1*, *COL6A3*, *COL1A1*, *COL13A1*, *SPP1*, and *MMP3*). RSG pretreatment also upregulated 21/40 (52.5%) “would healing” genes (Supplementary Fig. S7B).

**Table 3.** Differentially Expressed Genes in MIO-M1 Cells

Number of DE genes^a^		H_2_O_2_ vs. control			
		Up	Down	No change	Total
H_2_O_2_ + RSG vs. H_2_O_2_	Up	4	219	197	420
	Down	45	16	132	193
	No change	903	847		
	Total	952	1082		

^a^ Genes upregulated, downregulated, or not DE are labeled as *up*, *down*, or *no change*, respectively.

### Biological pathway enrichment analysis

Next, an analysis was done to identify the biological pathways that were overrepresented in the DE genes. In both cell models, 3 main groups of pathways were significantly enriched: integrin, AP-1, and syndecan signaling pathways (Table 4). In ARPE-19 cells, the beta1 integrin pathway was enriched with H_2_O_2_-regulated genes, while 3 integrin pathways (beta1, beta3, and integrins in angiogenesis), AP-1 transcription factor pathway, and 2 syndecan signaling pathways (syndecan 1 and 2) were enriched with RSG pretreatment-regulated genes. The beta1 integrin pathway was enriched by both analyses and showed the strongest statistical significance in the data set. In addition, a majority of pathway genes were downregulated by H_2_O_2_ and upregulated by RSG (Supplementary Table S1). The genes with the largest expression changes by RSG included *FOS*, *EGR1*, *CCL2*, and *CXCL8* in the AP-1 and syndecan pathways, and *COL4A1*, *COL5A1*, *THBS1*, *ITGA7*, and *MMP2* in the integrin and syndecan pathways.

**Table 4.** Biological Pathways Enriched with Differentially Expressed Genes in ARPE-19 and MIO-M1 Cells

Pathway	FDR	Number of genes^a^
ARPE-19 – H_2_O_2_ vs. Control		
Beta1 integrin cell surface interactions	0.0392	29
ARPE-19 – H_2_O_2_ + RSG vs. H_2_O_2_		
Beta1 integrin cell surface interactions	9.39E-10	17
Beta3 integrin cell surface interactions	1.86E-07	12
AP-1 transcription factor network	0.00851	9
Integrins in angiogenesis	0.00851	10
Syndecan-2-mediated signaling events	0.00851	7
Syndecan-1-mediated signaling events	0.00851	7
MIO-M1 – H_2_O_2_ vs. Control		
AP-1 transcription factor network	0.0134	22
Validated transcriptional targets of AP1 family members Fra1 and Fra2	0.0134	15
Integrins in angiogenesis	0.02	27
Beta1 integrin cell surface interactions	0.0367	24
MIO-M1 – H_2_O_2_ + RSG vs. H_2_O_2_		
Beta1 integrin cell surface interactions	0.000558	19
Syndecan-1-mediated signaling events	0.00525	13
Integrins in angiogenesis	0.00752	17
Beta3 integrin cell surface interactions	0.00883	11

^a^ The number of DE genes found in an enriched pathway.

FDR, false discovery rate.

In MIO-M1 cells, 2 AP-1 pathways (AP-1 transcription factor, and targets of AP1 family members Fra1 and Fra2) and 2 integrin pathways (beta1 and integrin in angiogenesis) were enriched with H_2_O_2_-regulated genes, while 3 integrin pathways (beta1, beta3, and integrins in angiogenesis) and syndecan-1 signaling pathway were enriched with RSG pretreatment-regulated genes. Integrins in angiogenesis and beta1 integrin pathways were enriched by both analyses. Similar to ARPE-19, a majority of regulated pathway genes were downregulated by H_2_O_2_ and upregulated by RSG (Supplementary Table S1). The genes with the largest expression changes by RSG included *COL1A1*, *COL6A3*, *COL4A4*, *COL12A1*, *COL9A3*, and *COL13A1* in the integrin and syndecan pathways, and *CSPG4*, *LAMA1*, *LAMA2*, and *SPP1* in the integrin pathways.

### Expression validation with qRT-PCR

RNA-seq revealed that H_2_O_2_ and RSG pretreatment regulated many genes in biological processes relevant to disease pathogenesis. To confirm RNA-seq findings, 12 RSG pretreatment-regulated genes each from ARPE-19 and MIO-M1 were analyzed by qRT-PCR with mRNA collected from cells processed for RNA-seq. The 12 genes included 6 genes involved in cell proliferation and death and 6 genes involved in ECM, cell adhesion, and cell migration (Table 1). For a majority of the genes (*CCN1*, *CCN2*, *EGR1*, *FAIM*, *FOS*, *HBEGF*, *NEDD9*, *SGK1*, and *THBS1* in ARPE-19 cells; *BACH2*, *BMF*, *COL1A1*, *COL6A3*, *EGR1*, *FMN1*, *MEGF10*, *MMP3*, *PGF*, *SGK1*, and *THSD7A* in MIO-M1 cells), there was high consistency in gene expression profiles between RNA-seq and qRT-PCR (Supplementary Figs. S5 and S6).

**FIG. 5.**
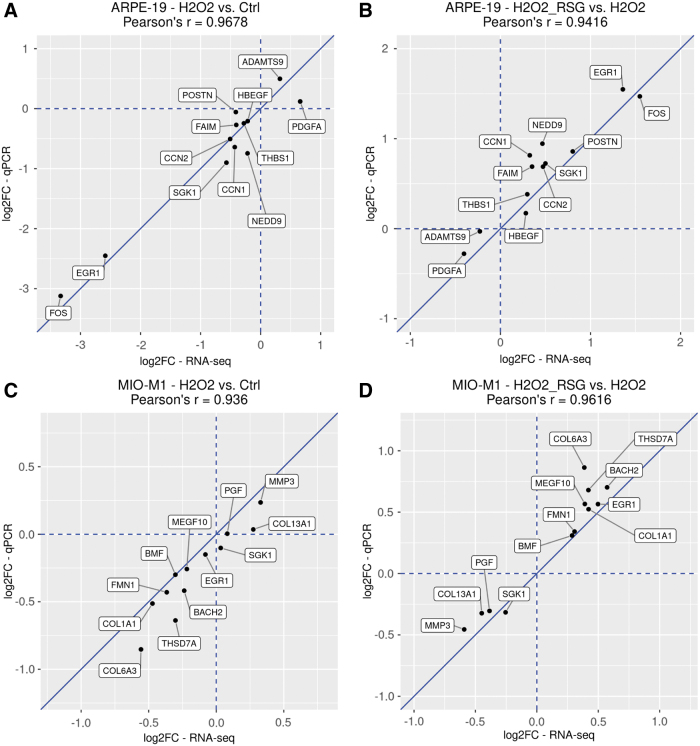
Expression fold change of selected genes analyzed by RNA-seq and qRT-PCR. RSG pretreatment-regulated genes identified through RNA-seq were selected for qRT-PCR validation. Fold change and Pearson's correlation values are visualized. ARPE-19 results are shown in **(A, B)**, and MIO-M1 results are shown in **(C, D)**. Fold changes associated with H_2_O_2_ regulation are shown in **(A, C)**, and fold changes associated with RSG pretreatment are shown in **(B, D)**. Log2-fold change (log2FC) values are plotted, with RNA-seq values on *x*-axis and qRT-PCR values on *y*-axis. *Blue dashed lines* represent the *x*- and *y*-axes, *blue solid lines* represent the *y* = *x* line, where the RNA-seq and qRT-PCR values are equal. The cell type, comparison tested, and Pearson's correlation values are shown in the title. qRT-PCR, quantitative real-time PCR.

Several genes (*ADAMTS9*, *PDGFA*, and *POSTN* in ARPE-19 cells; *COL13A1* in MIO-M1 cells) showed less consistent expression profiles between the measurement methods, but their direction of expression changes appears to be uniform. Indeed, in both ARPE-19 and MIO-M1 cells, strong correlations (*r* > 0.93) were observed between RNA-seq and qRT-PCR data (Fig. 5).

## Discussion

AMD and DR are major causes of blindness in developed nations and oxidative stress contributes to the disease development. In the affected retina, RPE and Müller cells are activated to provide an initial neuroprotective role, but eventually contribute to the development of pathological neovascularization, inflammation, and tissue degeneration.^14,20^ While anti-VEGF therapies are routinely used to treat neovascularization in a subset of patients, no therapeutics are approved for reduction of oxidative stress and retinal degeneration.^47^ This study used cell culture and transcriptomic approaches to study the effect of the oxidant H_2_O_2_ and the investigational drug RSG in human RPE (ARPE-19) and Müller (MIO-M1) cell lines.

H_2_O_2_ is a common by-product of cellular metabolism and is essential for cell growth at low levels.^48^ However, excess H_2_O_2_ is cytotoxic and is routinely used experimentally to study oxidative stress and cell death.^49^ H_2_O_2_ has shown to induce retinal cell apoptosis in both *in vitro*^50^ and *in vivo*^49^ settings. In this study, 12 h of H_2_O_2_ treatment significantly reduced cell viability in both ARPE-19 and MIO-M1 cells. In contrast, RSG had no detectable effect on viability of unstressed cells, while RSG pretreatment significantly reduced cell viability loss from H_2_O_2_. These findings corroborate earlier studies that found RSG protected human donor RPE cells damaged by H_2_O_2_ and a different toxin, hydroquinone.^24,51^

In addition, earlier work found that RSG pretreatment was much more effective in preventing cell death than post-treatment, perhaps because pretreatment prevents the cells from reaching a certain threshold the point at which they are committed to cell death and cannot be rescued.^52^ This also connects to the clinical setting where RSG improved vision in dry AMD patients with early disease stage, before significant and irreversible retinal damage has occurred.^22^ In the advanced stage of dry AMD (geographic atrophy), where retinal damage is extensive, 2 late-stage investigational drugs (Zimura and APL-2) can only slow down but not stop lesion growth.^53^ Thus, we combined drug pretreatment and RNA-seq to further investigate the regulatory dynamics involved in H_2_O_2_ cytotoxicity and protection by RSG.

Using a sufficiently large set of biological replicates, the RNA-seq data showed H_2_O_2_ exposure altered the expression of 3,686 genes in ARPE-19 and 2,034 genes in MIO-M1 cells. In contrast, the effect of RSG pretreatment was relatively small, involving 316 genes in ARPE-19 and 613 genes in MIO-M1 cells. When evaluated against untreated control, both cell models pretreated with RSG before H_2_O_2_ exposure had fewer DE genes than cells treated with H_2_O_2_ alone, suggesting RSG suppressed the effects of H_2_O_2_. This is supported by an analysis of H_2_O_2_-regulated genes that found RSG pretreatment effectively mitigated expression changes associated with H_2_O_2_ exposure.

A functional analysis of the regulated genes identified the biological processes and pathways affected by H_2_O_2_ exposure and RSG pretreatment. Intriguingly, many disease-relevant biological processes were regulated by both treatments, including cell adhesion, migration, death, and proliferation; ECM organization; angiogenesis; metabolism; immune system process; and others. Regulation of cell death and proliferation genes is consistent with the observed changes in the cell viability experiment. Elevated oxidative stress has also been associated with the development of retinal neovascularization,^54^ inflammation,^55^ and metabolic dysfunction.^56,57^ In addition, reorganization of ECM and dynamic changes in cell adhesion and migration are key cellular processes during retinal disease progression, such as during neovascularization and inflammation.^58,59^ A more detailed analysis found that while many of the same biological processes were coregulated, they have opposite gene expression profiles, further indicating that RSG treatment protected cells by suppressing the effects of oxidative stress.

Previous studies also found RSG beneficially regulated disease-relevant genes in RPE cells,^24,25^ but this is the first report of a similar behavior in a Müller cell line. To confirm the observed RNA-seq expression profiles, we used qRT-PCR to validate the expression of 12 genes each in ARPE-19 and MIO-M1 cells, selected for known function in ECM organization and cell adhesion, migration, death, and proliferation. Overall, strong positive correlations were detected between RNA-seq and qRT-PCR data, providing greater confidence in the whole transcriptome data set.

In the biological pathway analysis, 3 main groups of pathway were identified: integrin, AP-1, and syndecan signaling pathways. Similar to the biological process analysis, there was considerable overlap between the pathways affected by H_2_O_2_ and RSG pretreatment. Interaction between integrins and their ECM ligands influences most cellular functions, including cell adhesion, migration, and survival decisions.^60^ Under oxidative stress conditions, integrin trafficking and expression changes have been reported to regulate cell adhesion and survival.^60,61^ In the context of RSG biology, regulation of integrin signaling pathways is not surprising as the drug has been shown to modulate expression of integrin genes and integrin-associated processes such as cell adhesion and migration.^24,25^

However, regulation of AP-1 and syndecan pathways by RSG under oxidative stress condition has not previously been reported. The AP-1 transcription factor is known to play a critical role in regulating a diverse array of cellular response to pro-oxidant conditions, including activation of antioxidative processes, inflammation, and cell survival decision.^62,63^ Regulation of AP-1 pathways by RSG may be significant, as AP-1 has been shown to be involved in both angiogenesis and inflammatory signaling in the retina.^64^

Syndecans are a small family of membrane receptors known to interact with actin cytoskeleton, ECM, GF, intracellular kinases, and integrins to regulate cell survival, proliferation, adhesion, and angiogenesis.^65^ It has been documented that oxidative stress induces syndecan activation and is associated with immune chemotaxis, wound healing, and fibrogenesis in diseases.^66^ While our expression data suggest that RSG acts to minimize peroxide's effect on these pathways, the exact role it plays in regulating these pathways during elevated oxidative stress condition is unclear and warrants further investigation.

A vast amount of existing literature indicates that the pathophysiology of human retinal diseases is incredibly complex^67–69^ and cannot be captured by any single experimental model.^70–72^ The immortalized RPE and Müller cell lines used in the study are cultured *in vitro*, isolated from other relevant retinal cells, including photoreceptors, microglia cells, and endothelial cells, that play a role in disease pathophysiology. In addition, the ARPE-19 cells in this study were stressed at subconfluent density as it has previously been reported that high-density RPE cell culture, which better resembles a physiological RPE monolayer, is highly resistant to peroxide stress.^29^ Nonetheless, cell culture models are highly reproducible and can provide insight into the underlying disease mechanisms and potential therapeutic interventions. In this study, we demonstrated that oxidative stress-induced cell death in *in vitro* RPE and Müller cell lines is accompanied by profound transcriptome changes in disease-relevant processes.

These observations suggest that oxidative stress not only activates cell death but also modulates other pathways that could further exacerbate retinal dysfunction and degeneration. Indeed, the current literature supports the existence of positive regulation between oxidative stress, inflammation, and angiogenesis in AMD,^73^ DR,^74^ and other disorders.^75^ Given the important role oxidative stress has been recognized in the development of retinal diseases, multiple antioxidants have been tested in preclinical and clinical settings with varying levels of success.^53,76^ At present, the only accepted therapeutic intervention for dry AMD is a combination of antioxidant vitamins and minerals, but the beneficial effect is modest.^77^ For RSG intravitreal injection, 3 Phase 2 clinical trials demonstrated significant visual acuity improvement in patients with dry AMD and DME.^22,23^

Our current findings suggest RSG's therapeutic effect may involve favorably regulation of cell death, neovascularization, inflammation, and metabolic dysfunction, which are involved in the pathophysiology of the clinically studied retinal diseases.^24,25^ These results expand our understanding of RSG's mechanism of action and support further clinical trials in the currently targeted indications and other pathologically relevant retinal diseases such as retinitis pigmentosa and glaucoma. More broadly, these pathological mechanisms are present in many human diseases, where RSG may potentially serve as a novel therapeutic option.

## Conclusions

In summary, we found RSG has a cytoprotective effect in ARPE-19 and MIO-M1 cells against H_2_O_2_-induced cell death. Whole transcriptome analysis showed genes in many disease-relevant biological processes (including cell adhesion, migration, death, and proliferation; ECM organization; angiogenesis; metabolism; and immune system processes) were regulated by both H_2_O_2_ exposure and RSG pretreatment, but in opposite directions. Pathway analysis showed novel insight that under oxidative stress conditions, RSG regulated integrin, AP-1, and syndecan pathways. Altogether, our results suggest RSG exhibits cytoprotective properties in cells under oxidative stress, which could lead to novel therapy for retinal degenerative diseases.

## Supplementary Material

Supplemental data

Supplemental data

Supplemental data

Supplemental data

Supplemental data

Supplemental data

Supplemental data

Supplemental data

Supplemental data
