# Supplementary material for: The Transcriptome Profile of Retinal Pigment Epithelium and Müller Cell Lines Protected by Risuteganib Against Hydrogen Peroxide Stress
Source: J Ocul Pharmacol Ther. 2022 Sep 12;38(7):513–26. doi: 10.1089/jop.2022.0015 (PMC9508878; doi:10.1089/jop.2022.0015)
Supplement: Supplemental data [file Supp_FigS8.docx]

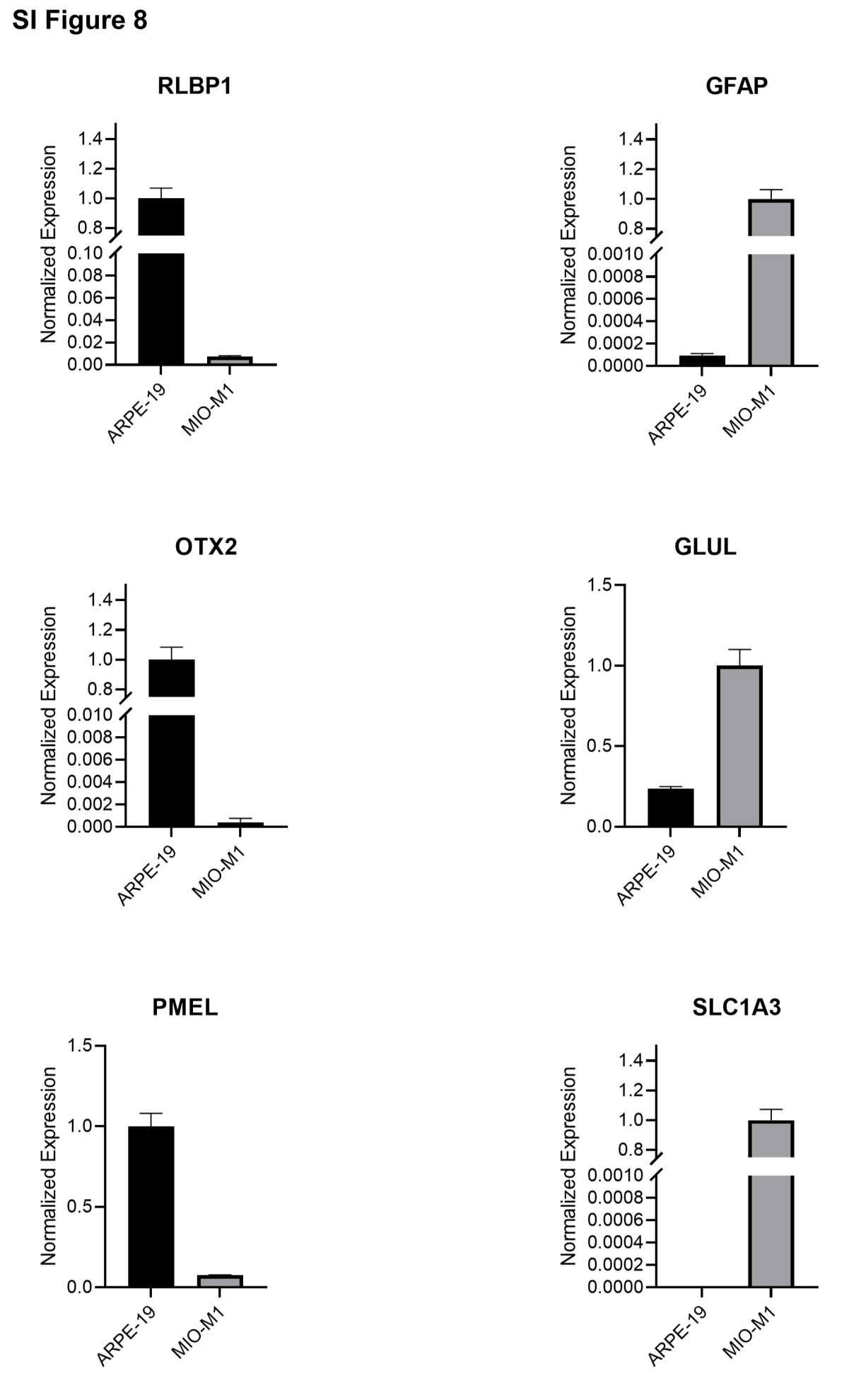


**S8 Fig. qRT-PCR expression profile of RPE and Müller cell markers**

Expression level (mean ± standard error of mean) measured by qRT-PCR in ARPE-19 (n=6) and MIO-M1 (n=6) cells are shown. Expression values are normalized to mean of ARPE-19 samples for *RLBP1*, *OTX2*, and *PMEL* and mean of MIO-M1 samples for *GFAP*, *GLUL*, and *SLC1A3*. *SLC1A3* expression in ARPE-19 samples are shown as zero since it was below the limit of detection.
