# Supplementary material for: The Transcriptome Profile of Retinal Pigment Epithelium and Müller Cell Lines Protected by Risuteganib Against Hydrogen Peroxide Stress
Source: J Ocul Pharmacol Ther. 2022 Sep 12;38(7):513–26. doi: 10.1089/jop.2022.0015 (PMC9508878; doi:10.1089/jop.2022.0015)
Supplement: Supplemental data [file Supp_FigS1.docx]

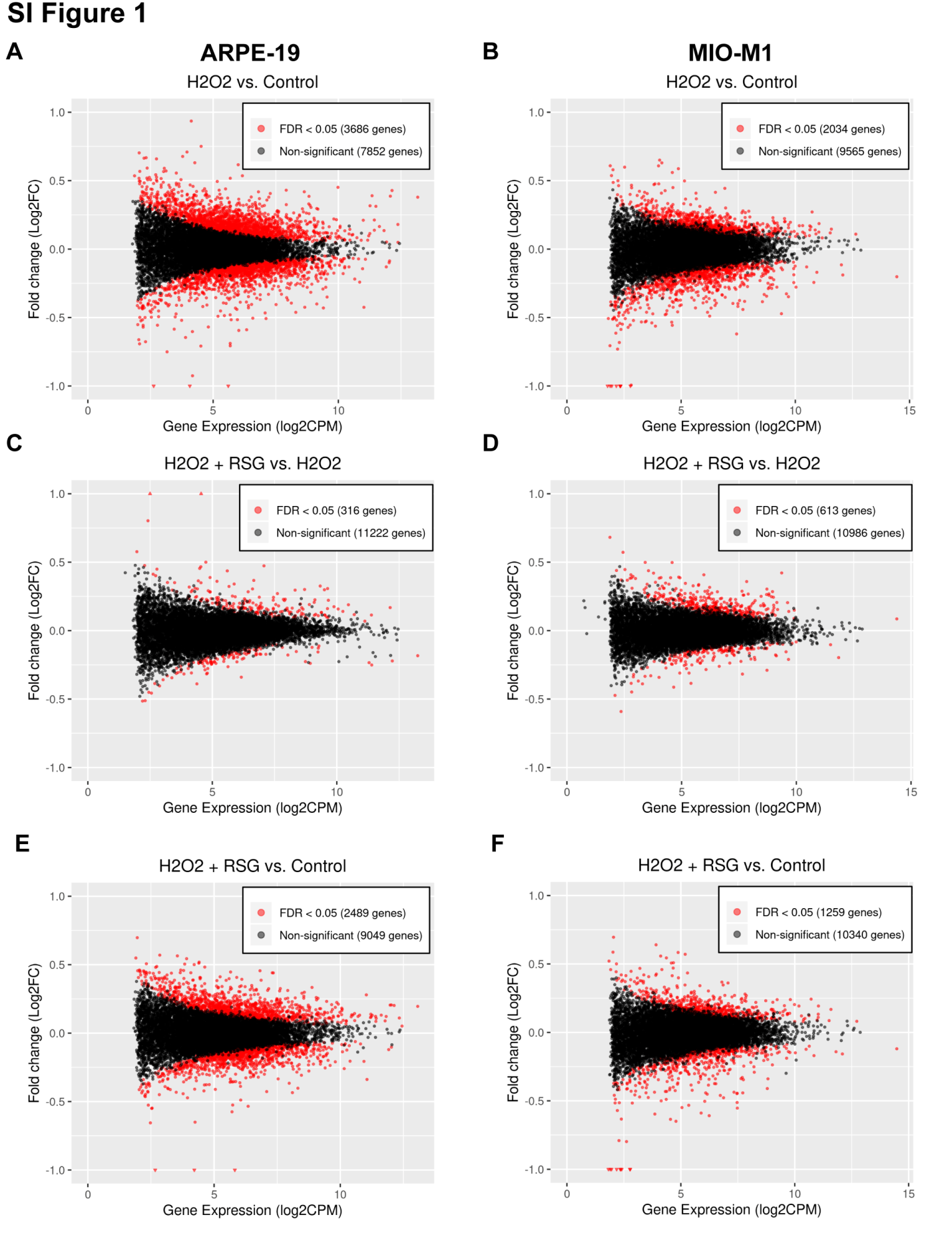


**S1 Fig. Statistics of differential expression comparisons.**

For each comparison in ARPE-19 (A, C, E) and MIO-M1 (B, D, F) cells, all expressed genes’ expression level (log2CPM) and fold change (log2FC) are visualized. DE genes are shown in red, non-DE genes are shown in black. Display is limited to (-1, 1) range on Y-axis; genes outside of display window are represented by solid triangles. Relatively few DE genes were modified by RSG pre-treatment, while H_2_O_2_ exposure generated large transcriptome changes.
