# Supplementary material for: The Transcriptome Profile of Retinal Pigment Epithelium and Müller Cell Lines Protected by Risuteganib Against Hydrogen Peroxide Stress
Source: J Ocul Pharmacol Ther. 2022 Sep 12;38(7):513–26. doi: 10.1089/jop.2022.0015 (PMC9508878; doi:10.1089/jop.2022.0015)
Supplement: Supplemental data [file Supp_FigS2.docx]

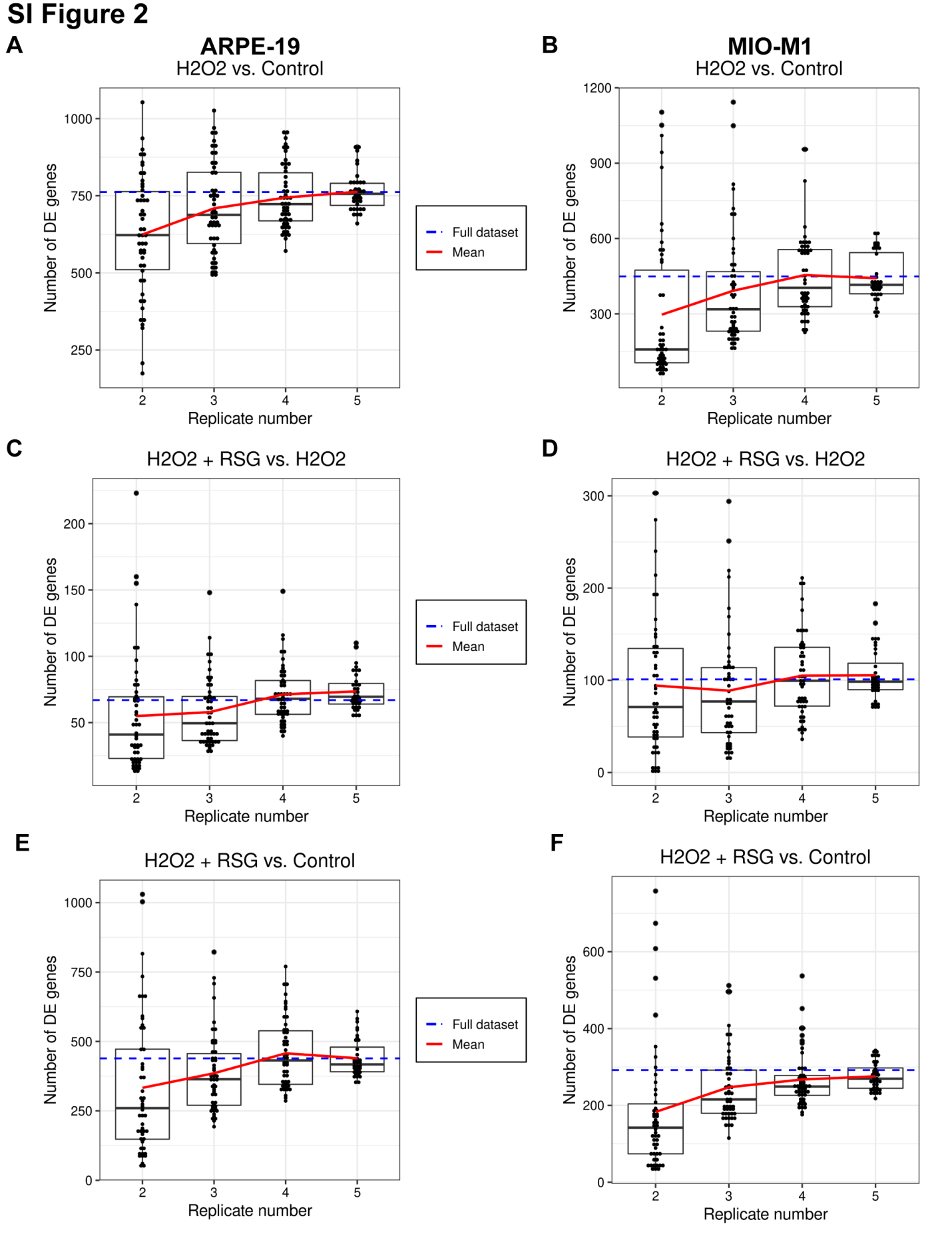


**S2 Fig. RNA-seq sample size analysis.**

ERSSA software was used to confirm sufficient biological replicates were employed for each of the differential expression comparisons in ARPE-19 (A, C, E) and MIO-M1 (B, D, F) cells. For all comparisons, the marginal increase in DE gene discovery became progressively smaller as the sample size increases. The marginal improvements were too small to warrant addition of more replicates to further enhance discovery.
