# Supplementary material for: The Transcriptome Profile of Retinal Pigment Epithelium and Müller Cell Lines Protected by Risuteganib Against Hydrogen Peroxide Stress
Source: J Ocul Pharmacol Ther. 2022 Sep 12;38(7):513–26. doi: 10.1089/jop.2022.0015 (PMC9508878; doi:10.1089/jop.2022.0015)
Supplement: Supplemental data [file Supp_FigS3.docx]

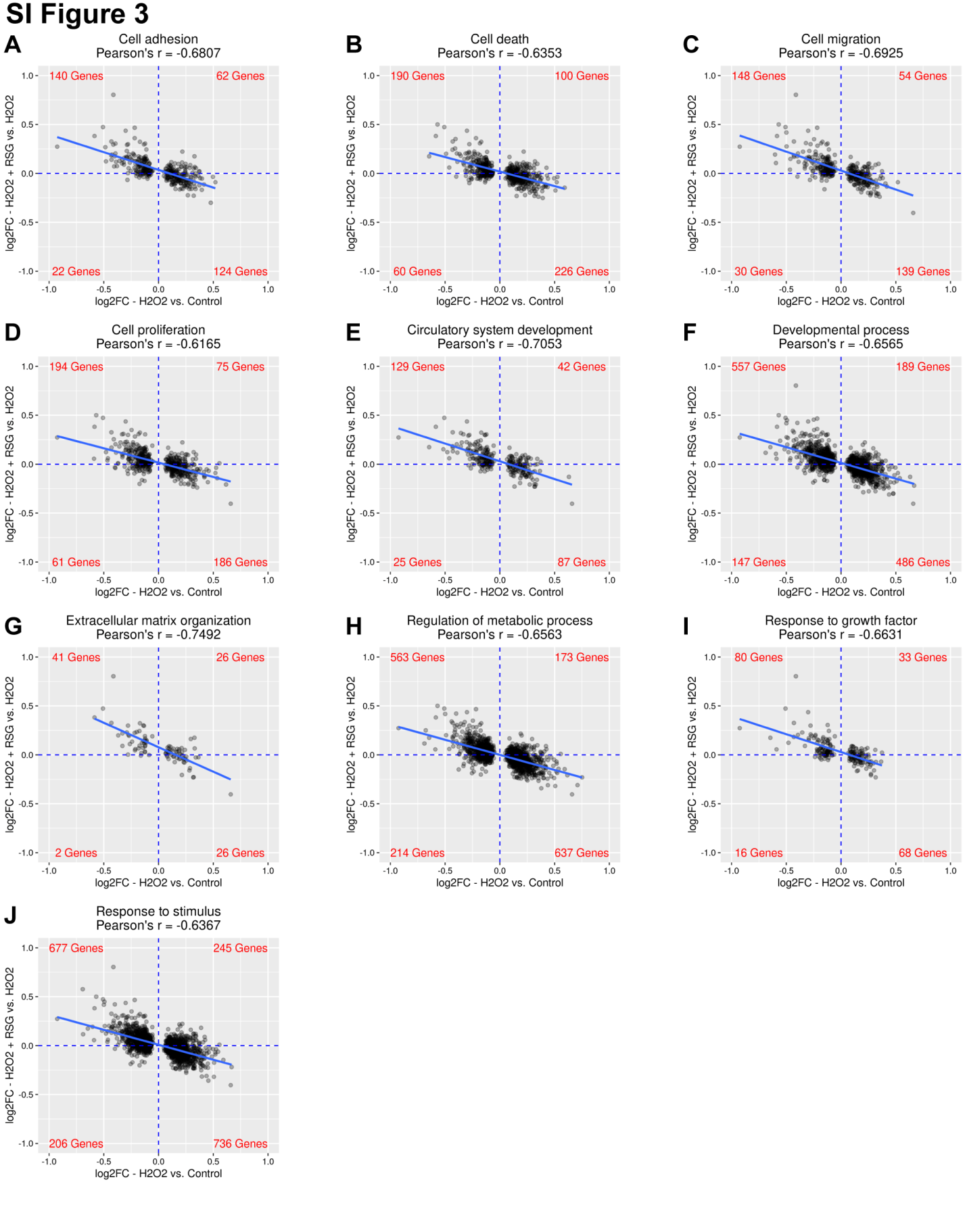


**S3 Fig. Biological processes commonly modulated by H_2_O_2_ and RSG pre-treatment in ARPE-19 cells.**

The selected biological processes represent category of processes commonly enriched with genes regulated by either H_2_O_2_ exposure or RSG pre-treatment in ARPE-19 cells. Fold change (log2FC) of H_2_O_2_-regulated genes in each of the processes are visualized. X-axis represents the gene’s log2FC as regulated by H_2_O_2_, Y-axis represents the corresponding log2FC as regulated by RSG pre-treatment. Display is limited to (-1.0, 1.0) range on both axes. Linear regression of data is shown as solid blue lines. Blue dashed lines separate the data into four quadrants with the number of genes in each quadrant labeled at the corners in red. Negative Pearson’s correlation coefficients were observed between expression changes associated with H_2_O_2_ exposure and RSG pre-treatment for each of the biological processes.
