# Supplementary material for: The Transcriptome Profile of Retinal Pigment Epithelium and Müller Cell Lines Protected by Risuteganib Against Hydrogen Peroxide Stress
Source: J Ocul Pharmacol Ther. 2022 Sep 12;38(7):513–26. doi: 10.1089/jop.2022.0015 (PMC9508878; doi:10.1089/jop.2022.0015)
Supplement: Supplemental data [file Supp_FigS7.docx]

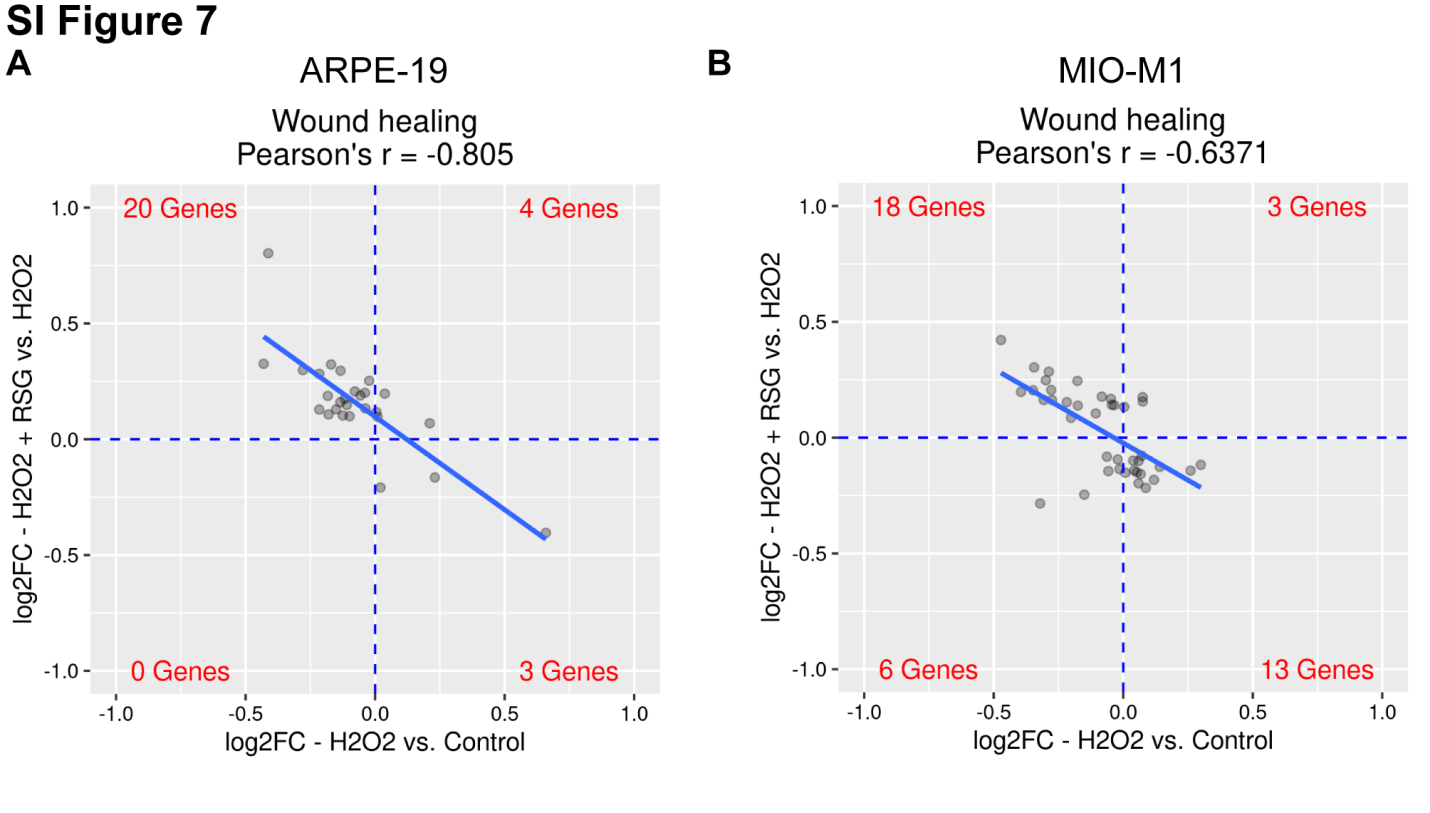


**S7 Fig. Wound healing genes regulated by RSG pre-treatment.**

“Wound healing” genes that were regulated by RSG pre-treatment in (A) ARPE-19 or (B) MIO-M1 cells are evaluated. Fold change (log2FC) are visualized. X-axis represents the gene’s log2FC as regulated by H_2_O_2_, Y-axis represents the corresponding log2FC as regulated by RSG pre-treatment. Display is limited to (-1.0, 1.0) range on both axes. Linear regression of data is shown as solid blue lines. Blue dashed lines separate the data into four quadrants with the number of genes in each quadrant labeled at the corners in red.
