# Supplementary material for: The Transcriptome Profile of Retinal Pigment Epithelium and Müller Cell Lines Protected by Risuteganib Against Hydrogen Peroxide Stress
Source: J Ocul Pharmacol Ther. 2022 Sep 12;38(7):513–26. doi: 10.1089/jop.2022.0015 (PMC9508878; doi:10.1089/jop.2022.0015)
Supplement: Supplemental data [file Supp_FigS6.docx]

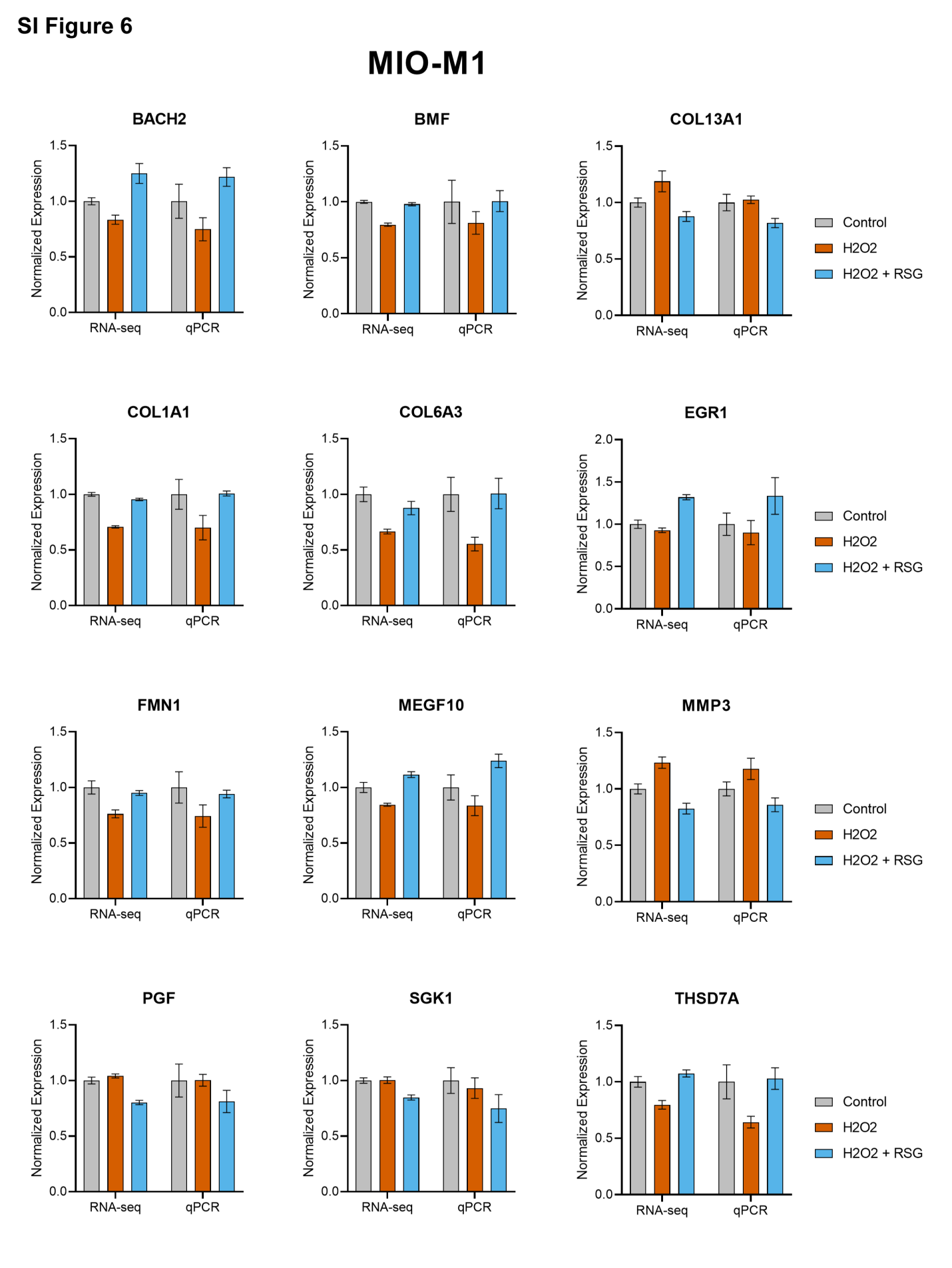


**S6 Fig. qRT-PCR expression profile of selected genes in MIO-M1 cells.**

Expression level (mean ± standard error of mean) measured by RNA-seq and qRT-PCR are shown. Expression values are normalized to mean of control.
